# Work-relatedness of lung cancer by smoking and histologic type in Korea

**DOI:** 10.1186/s40557-014-0043-y

**Published:** 2014-12-01

**Authors:** Young-Il Lee, Sang-Gil Lee, Dong-Mug Kang, Jong-Eun Kim, Young-Ki Kim, Jong-Han Leem, Hwan-Cheol Kim

**Affiliations:** Department of Occupational and Environmental Medicine, Pusan National University, Yangsan Hospital, Yangsan, 626-770 South Korea; Occupational Safety and Health Research Institute, Korea Occupational Safety and Health Agency, Jongga-ro 400, Jung-gu, Ulsan 681-230 Republic of Korea; Department of Occupational and Environmental Medicine, Inha University Hospital, Incheon, South Korea

**Keywords:** Occupational lung cancer, Work-relatedness, Histologic type

## Abstract

**Objectives:**

This study investigated the distribution of causative agents related to occupational lung cancer, their relationships with work, and associations between work-relatedness and the histologic type of lung cancer.

**Methods:**

We used data from the occupational surveillance system in Korea in 2013. In addition, data from 1,404 participants diagnosed with lung cancer were collected through interviews. We included the patients’ longest-held job in the analysis. Work-relatedness was categorized as “definite,” “probable,” “possible,” “suspicious,” “none,” or “undetermined.”

**Results:**

Among the subjects, 69.3% were men and 30.7% were women. Regarding smoking status, current smokers were the most prevalent (35.5%), followed by non-smokers (32.3%), ex-smokers (32.2%). Regarding the causative agents of lung cancer, asbestos (1.0%) and crystalline silica (0.9%) were the most common in definite work-related cases, while non-arsenical insecticide (2.8%) was the most common in probable cases followed by diesel engine exhaust (1.9%) and asbestos (1.0%). Regarding histologic type, adenocarcinoma was the most common (41.7%), followed by squamous cell carcinoma (21.2%). Among current smokers, squamous cell carcinoma was the most common among definite and probable cases (13.4%), while non-small cell lung cancer was the least common (7.1%). Among non-smokers, squamous cell carcinoma was the most common (21.4%), while the least common was adenocarcinoma (1.6%).

**Conclusions:**

Approximately, 9.5% of all lung cancer cases in Korea are occupational-related lung cancer. Well-known substances associated with lung cancer, such as crystalline silica, asbestos, and diesel engine exhaust, are of particular concern. However, the histologic types of lung cancer related to smoking were inconsistent with previous studies when work-relatedness was taken into account. Future studies are required to clarify the incidence of occupational lung cancer in agricultural workers exposed to non-arsenical insecticides and the associations between work-relatedness and the histologic type of lung cancer.

## Introduction

The epidemiology of occupational cancer varies by country. However, approximately 2–8% of all cancer deaths are related to occupational exposure [1]. The National Cancer Information Center estimates the burden of cancers attributable to occupational exposure in Korea is 9.7% [2]. However, further investigation considering occupational history is required to produce more precise estimates. Occupational lung cancer is the most burdensome type of cancer owing to its relatively high mortality.

However, most studies using cancer registry data to analyze occupational cancer tend to be passive, and few studies have utilized nationally representative data related to occupational lung cancer.

While there are many studies about the relationship between the histologic type of lung cancer and smoking [3,4], little is known about the relationship between the histologic type of lung cancer. Moreover, most existing studies only examined associations with occupations and identified exposure to causative agents and histologic type [5,6]. Therefore, studies investigating the association between occupation and exposure levels are needed to clarify this association.

Adequate management of occupational hazardous substances can reduce the incidence of occupational disease. Many developed countries that have acknowledged the occupational disease are actively trying to reduce the incidence of occupational disease through various means such as occupational surveillance systems [7]. Various occupational surveillance studies have been performed in Korea. However, most are limited to certain regions and studies about occupational cancer are particularly insufficient [8-11]. Furthermore, it is difficult to estimate precisely exposure levels of causative agents. Moreover, the diagnostic rate of occupational cancer is lower than that of other occupational diseases because of its long latency.

Accordingly, this study investigated the distribution of causative agents related to lung cancer, their relationship with work, and associations between work-relatedness and histologic type of lung cancer based on the occupational surveillance data of 2013 in Korea

## Methods

### Participating hospitals

**Figure 1:**
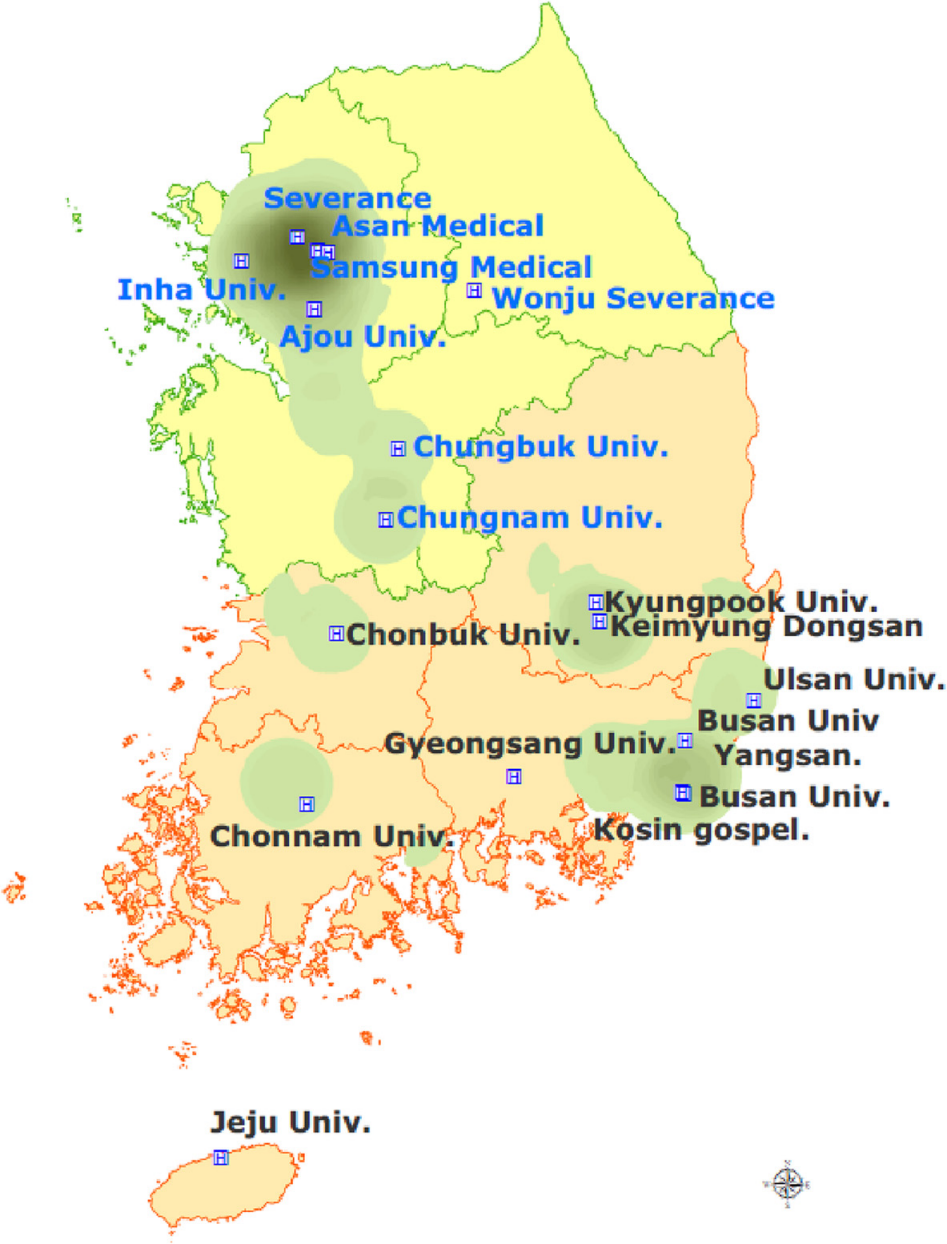
**Participating hospitals.**

The data sources were divided into middle and southern regions of Korea, and trained investigators conducted interviews with lung cancer patients selected from each region. We stratified into 16 administrative regions consisting of metropolitan city and province across the country, and each metropolitan city and province was defined as one independent unit; in each area, we selected regional hub hospitals that are established cancer centers because each cancer patients would be likely to be concentrated around regional hub hospitals. For the large hospitals of capital area, we selected multiple hospitals because these are representative not only in capital area but also in nationwide region. Finally, 8 and 10 hospitals in the middle and southern areas were selected. This study was approved by the respective institutional review board of each hospital, and all patients provided informed consent prior to enrollment (Figure 1).

### Study population

The patients were recruited from January to August 2013. Only patients aged 20 year or older among patients newly diagnosed or histopathologically confirmed to be lung cancer were included. Patients with prior chemotherapy or radiotherapy for other cancers including lung cancer were excluded. The histologic type of lung cancer was classified as small cell lung cancer (SCLC), non-small cell lung cancer (NSCLC) which includes adenocarcinoma (ADC), squamous cell carcinoma (SCC), large cell carcinoma (LCC); bronchogenic carcinoma and ambiguous types were classified as others. After informed consent was obtained each patient, professionally trained interviewers in each participating hospital administered a structured questionnaire through in-person or telephone interviews to collect information on demographic data; as well as other risk factors for lung cancer, including smoking status, family history, and occupational history. Non-smokers were defined as those who had smoked less than 100 cigarettes in their lifetime [12] and ex-smokers were defined as those who had quit smoking more than 6 months before diagnosis. The occupational questionnaire included monthly workdays, protective equipment use, daily working time, work process, and handling of hazardous substances. The type of occupation was recorded in the order of longest-held job, current, and past job; only the longest-held job was used in the final analysis.

### Case definition and evaluation of work-relatedness

Standardized criteria for defining cases of occupational lung cancer and assessing work-relatedness were developed. Work-related causative agents and work processes were reclassified on the basis of a literature review of the International Agency for Research on Cancer (IARC). The case definition was evaluated considering diagnostic accuracy (i.e., histologic confirmation), work-related carcinogen exposure (IARC group 1 and 2A), latency, degree of exposure taking into account exposure intensity, daily exposure duration, and total exposure duration. We considered that exposed period is sufficiently enough if it is more than 10 years. Although the exposed period is less than 10 years, we evaluated work-relatedness considering degree of cumulative exposure. The latency period of lung cancer for evaluation was considered 10 years and all data collected from interviews were consolidated into a database. The judgment of “work-relatedness” was discussed with occupational, environmental, and industrial hygiene specialist and was ultimately categorized “definite”, “probable”, “possible”, “suspicious”, “none”, or “undetermined”.

### Statistical analyses

Descriptive statistical analyses were first conducted to characterize the study population. Age, smoking history, histologic type of lung cancer, and work-relatedness are expressed as frequencies and percentages. Their associations with sex were analyzed by using the *χ*^2^ test and Fisher’s exact test where appropriate. In addition, the distribution of causative agents with respect to work-relatedness was analyzed, and the association between work-relatedness and histologic type stratified by smoking status was determined by using Cochran-Mantel-Haenszel test. The level of significance was set at *p* < 0.05. All analyses were performed by using SAS version 9.2 (SAS Institute Inc., Cary, NC, USA).

## Results

### Patient characteristics

The patients’ characteristics are shown in Table 1. A total of 1,404 patients were analyzed; 69.3% were men, and 30.7% were women. The mean age of all patients was 64.6 years old; men (mean age: 65.5 years) were significantly older than women (mean age: 62.7 years, *p* < 0.001). Regarding age distribution, patients aged >70 years were prominent (35.6%). Age distribution was similar in each sex. Regarding smoking status, current smokers were the most prevalent (35.5%), followed by non-smokers (32.3%), ex-smokers (32.2%). When stratified according to sex, mean showed similar trends, but 86.8% of women reported being “non-smokers”. Regarding the histologic type of lung cancer in all patients, ADC was prominent (41.7%). By sex, ADC (31.8%) and SCC (28.1%) were the most common in men, while ADC (64.3%) was the most common in women. Regarding work-relatedness, definite cases were 2.2% and probable cases were 7.3%. In all patients, 9.5% of cases were probable or definite; definite cases were only recorded in men.

**Table 1:** **General characteristics of the study subjects** (**N** = **1**,**404**)

**Characteristics**	**Men** **(** **n** **=** **973** **)**		**Women** **(** **n** **=** **431** **)**		**p value**
	***n***	**%**	***n***	**%**	
Age					p < 0.001^*^
20 ~ 29	3	0.3	3	0.7	
30 ~ 39	7	0.7	8	1.9	
40 ~ 49	52	5.3	39	9.0	
50 ~ 59	186	19.1	118	27.4	
60 ~ 69	356	36.6	132	30.6	
≥70	369	37.9	131	30.4	
Smoking history					p < 0.001^**^
Non-smoker	80	8.2	374	86.8	
Ex-smoker	424	43.6	28	6.5	
Current smoker	469	48.2	29	6.7	
Histologic type					p < 0.001^**^
SCLC	130	13.3	17	3.9	
NSCLC					
ADC	309	31.8	277	64.3	
SCC	273	28.1	24	5.6	
LCC	30	3.1	10	2.3	
Others^†^	231	23.7	103	23.9	
Work-relatedness					p < 0.001^**^
Definite	31	3.2	0	0.0	
Probable	95	9.8	7	1.6	
Possible	296	30.4	44	10.2	
Suspicious	194	19.9	63	14.6	
No relation	354	36.4	317	73.6	
Undetermined	3	0.3	0	0.0	

SCLC: Small cell lung cancer.

NSCLC: Non-small cell lung cancer.

ADC: Adenocarcinoma.

SCC: Squamous cell carcinoma.

LCC: Large cell carcinoma.

^†^bronchogenic carcinoma and undetermined.

^*^Fisher’s exact test.

^**^ *χ* ^2^ test.

### Work-relatedness of causative agents

On the basis of occupational history, a total of 24 occupational-related hazardous agents or processes were ascertained. The work-relatedness of each of causative agent is shown in Table 2. Crystalline silica, nickel, radon, asbestos, and foundry were associated with definite cases; asbestos (1.0%) was the most common, followed by crystalline silica (0.9%). Among probable cases, non-arsenical insecticides (2.8%) was the most common, followed by diesel engine exhaust (1.9%) and asbestos (1.0%). Among definite and probable cases, non-arsenical insecticide (2.8%) was the most common causative agent, followed by asbestos (2.0%), diesel engine exhaust (1.9%), and crystalline silica (1.3%). When stratified by sex, non-arsenical insecticide was the most common in both sexes, but there were no definite cases in women (data not shown).

**Table 2:** **Work-relatedness with respect to causative agent**

**Work-** **relatedness**	**Substance**	**Men**		**Women**		**Total**	
		***n***	**%**	***n***	**%**	***n***	**%**
Definite	Asbestos	14	1.4	0	0.0	14	1.0
	Crystalline silica	12	1.2	0	0.0	12	0.9
	Foundry	2	0.2	0	0.0	2	0.1
	Nickel	1	0.1	0	0.0	1	0.1
	Painting	1	0.1	0	0.0	1	0.1
	Radon	1	0.1	0	0.0	1	0.1
Probable	Non-arsenical insecticides	34	3.5	5	1.2	39	2.8
	Diesel engine exhaust	27	2.8	0	0.0	27	1.9
	Asbestos	14	1.4	0	0.0	14	1.0
	Crystalline silica	5	0.5	0	0.0	5	0.4
	Welding fume	4	0.4	0	0.0	4	0.3
	Painting	3	0.3	0	0.0	3	0.2
	Hexavalent chromium	3	0.3	0	0.0	3	0.2
	Printing process	2	0.2	0	0.0	2	0.1
	TCDD^*^	1	0.1	0	0.0	1	0.1
	Soot	1	0.1	0	0.0	1	0.1
	Asphalt work	1	0.1	0	0.0	1	0.1
	High temperature frying	0	0.0	2	0.5	2	0.1
Others^†^		847	87.2	424	98.3	1271	90.4
	Total	973	100.0	431	100.0	1404	100.0

*TCDD: 2,3,7,8-tetrachlorodibenzopara-dioxin.

^†^Possible, suspicious, no relation, or undetermined cases.

### Histologic type and work-relatedness

The association between histologic type and work-relatedness according to smoking status is shown in Table 3. Regarding smoking status, SCC was the most common type of lung cancer both in smokers (13.4%) and non-smokers (21.4%) including ex-smokers. After adjusting for smoking, similar trends were observed, with SCC being the most common (13.8%).

**Table 3:** **Work**-**relatedness by histologic type according to smoking status**

**Histology**	**Smoking** ^*^				**Non**-**smoking** ^**^				**Total** ^***^			
	**Work** **-** **relatedness** **(+)** ^**+**^		**Work** **-** **relatedness** **(-)** ^**++**^		**Work** **-** **relatedness** **(+)** ^**+**^		**Work** **-** **relatedness** **(-)** ^**++**^		**Work** **-** **relatedness** **(+)** ^**+**^		**Work** **-** **relatedness** **(-)** ^**++**^	
	**N**	**%**	**N**	**%**	**N**	**%**	**N**	**%**	**N**	**%**	**N**	**%**
SCLC	16	12.2	115	87.8	1	6.3	15	93.8	17	11.6	130	88.4
NSCLC												
ADC	26	9.2	256	90.8	5	1.6	299	98.4	31	5.3	555	94.7
SCC	38	13.4	245	86.6	3	21.4	11	78.6	41	13.8	256	86.2
LCC	2	7.1	26	92.9	2	16.7	10	83.3	4	10.0	36	90.0
Others	33	14.6	193	85.4	7	6.5	101	93.5	40	12.0	294	88.0
total	115	12.1	835	87.9	18	4.0	436	96.0	133	9.5	1271	90.5

NSCLC: non-small cell lung cancer, ADC: adenocarcinoma, SCLC: small cell lung cancer, SCC: squamous cell carcinoma, LCC: Large cell carcinoma.

Others: bronchogenic carcinoma and undetermined.

^*^p = 0.0346, *χ* ^2^ test.

^**^p = 0.0004, Fisher’s exact test.

^***^p = 0.0421, Cochran–Mantel–Haenszel test.

^+^Definite and probable cases.

^++^Possible, suspicious, no relation, and undetermined cases.

## Discussion

This study estimated the proportion of occupational lung cancer among all lung cancers in Korea to be 9.5%. A previous study provides an estimate of 5.3%, and the attributable fraction of lung cancer was 14.3%, behind mesothelioma and sino-nasal cancer [13]. Meanwhile, Driscoll et al. [14] estimate the proportions of occupational lung cancer to be 10% in men and 5% in women. Thus the present result is comparable with those of previous studies. However, the present results may be more accurate, considering that this study was a direct survey and not retrospective in nature.

Among the causative agents of lung cancer in the present study, the definite cases involved asbestos, crystalline silica, foundry, nickel, and non-arsenical insecticides. If probable cases are included, diesel engine exhaust and welding fume are also included. When considering only definite cases, the work-relatedness of asbestos was the second highest in total and the most frequent. LaDou estimates the occurrence of occupational lung cancer due to asbestos exposure to be 5–7% [15] compared to just 2% in the present. Lung cancer caused by asbestos exposure has a widely accepted latency time of 15–30 years [16]. Although asbestos has been banned in Korea since 2009 [17], the prevalence of asbestos-induced lung cancer is expected to increase in the future, because its occurrence has not peaked [18]. The association between crystalline silica exposure and lung cancer was controversial until the mid-1990s. However, at present, crystalline silica is known to be causatively related to lung cancer [19]. Accordingly, it was the second most common cause of definite cases in the present study. Diesel engine exhaust was previously classified into IARC group 2A, because evidence of its carcinogenicity was limited; however, it was reclassified as a group 1 carcinogen in 2012 [20]. In the present study, there were no definite cases related to diesel engine exhaust, but diesel engine exhaust was the second most common cause among probable cases. Regarding work-relatedness, non-arsenical insecticide was the most frequently reported among causative agents. Non-arsenical insecticides are classified as IARC group 2A to 3 according to the type [21]. Blair et al. [22] examined the health effects of chronic exposure to insecticides and report that the standardized mortality ratio for lung cancer is 135 and increases in a duration-dependent manner. The cohort study of Alavanja et al. [23] shows an increased risk of lung cancer with increasing lifetime use of insecticides after adjusting for smoking; however, this association varies depending on the types of insecticides. Non-arsenical insecticides are used mostly in fruit farms, the cultivation of flowers, and highland agriculture, but infrequently used in rice and upland farming. The US Environmental Protection Agency sets standards for the annual number of sprayings and application amounts as well as guidelines for cumulative risk assessment for the use of agricultural chemicals including insecticides [24]. However, these recommendations and guidelines have limited applicability in Korea because of differences in insecticide use. Moreover, it is difficult to accurately investigate insecticide exposure because farmers usually apply mixtures of 2 or more insecticides simultaneously and often forget which ones they applied. In addition, the differences in the method of insecticide application must be considered. The present study evaluated the work-relatedness of lung cancer focusing on the kinds of the agriculture that frequently use insecticides while considering the number of applications and exposure duration. The present nationwide study elucidated potential insecticide exposure, which has been largely overlooked until now. Regardless, additional epidemiological studies are required to more precisely determine the extent to which lung cancer is associated with agricultural work.

The histologic type of lung cancer is generally classified as small cell or non-small cell lung cancer, which includes ADC and SCC. SCC is more closely associated with smoking [25] and used to be the most frequent type of lung cancer. However, ADC has overtaken SCC, because cigarettes are now generally low-tar filtered cigarettes [26]. In the present study, ADC was the most common type (41.7%) followed by SCC (21.2%). The analysis between histologic type and work-relatedness showed the relative proportions of work-related cases of SCC and ADC were high and low, respectively.

The results show smoking status affects the work-relatedness of different lung cancer types. The proportion of work-related cases of ADC was high among current smokers, whereas that of SCC was high among non-smokers. In contrast, ADC is reported to primarily occur in non-smokers, whereas SCC is closely associated with smoking. Thus, the results suggest the existence of a combined effect between causative agents and smoking. Nevertheless, as the present cross-sectional study cannot determine causation, additional well-designed studies are required to clarify the association between the histologic type of lung cancer and smoking while considering occupational exposure. It is generally known that occupational lung cancer has no specific histologic type with respect to occupational carcinogens [27], but it is known to be associated with kinds of occupations. Zahm et al. [28] report that ADC is observed more frequently in plumbers and printers whereas SCC are more common in welders, although these differences are not statistically significant. Meanwhile, Elci et al. [5] report that work in textiles, grain milling, and construction are significantly associated with excess risks of SCC and ADC. MacArthur et al. [6] observed an increased risk of SCC in workers involved in pipeline transport, construction, and metal fabrication, as well as increased risk of ADC in those in field crop farming, machinery and equipment, and insulation work. Hence, additional studies are required to clarify the biological effects of various causative agents according to histologic type as well as the association between work-relatedness and histology according to occupational exposure level, because most published studies merely investigated the associations between occupations and the histologic type of lung cancer.

This study has some limitations. One limitation concerns the representativeness of the study sample. We compared the regional distribution of 2006–2009 average lung cancer patients of investigation through the national cancer registry data with that of occupational surveillance data of 2012, and identified that the distribution was similar. Therefore, we think that it is reasonable to use the regional distribution data, and we tried to select regional hub hospitals that cover lung cancer patients in their respective areas to ensure the data were representative. In the future, however, more precisely designed epidemiologic studies are required to confirm the present findings because the patients who were diagnosed at local hospitals tended to be concentrated at hospitals near the Seoul. Furthermore, there may be regional differences associated with case definitions or evaluation for work-relatedness among published surveillance studies in Korea. We clarified the standard for evaluating work-relatedness and defining occupational lung cancer cases through expert discussion. Because most of the study patients had 2 or more occupations, we ultimately evaluated work-relatedness on the basis of their longest-held. Accordingly, future studies must consider not only workers who have had many kinds of jobs, but also exposure to multiple occupational carcinogens.

Despite these limitations, the main strengths of this study are that it is an active and nationwide study. Compared to other surveillance studies based on cancer registry data, the present data are from direct surveys administered by professionally trained interviewers.

## Conclusions

In conclusion, the estimated the occurrence of occupational lung cancer in Korea among all lung cancers is 9.5%, which is not insubstantial. Well-known substances previously associated with the lung cancer such as crystalline silica, asbestos, and diesel engine exhaust are of specific concern. Future studies should specifically investigate the incidence of occupational lung cancer in agricultural workers according to exposure to non-arsenical insecticides. Furthermore, the histologic types of lung cancer related to smoking were inconsistent with previous studies when work-relatedness was taken into account. Future studies should determine the precise associations among the causes of lung cancer such as occupational hazardous agents and smoking, and histologic types.

## References

[1] Doll R, Peto R (1981). The causes of cancer: quantitative estimates of avoidable risks of cancer in the United States today. J Natl Cancer Inst.

[2] **National Cancer Information Center:** [http://www.cancer.go.kr/mbs/cancer/subview.jsp?id=cancer_010108010000]

[3] Janssen-Heijnen ML, Coebergh JW (2003). The changing epidemiology of lung cancer in Europe. Lung Cancer.

[4] Papadopoulos A, Guida F, Leffondré K, Cénée S, Cyr D, Schmaus A, Radoï L, Paget-Bailly S, Carton M, Menvielle G, Woronoff AS, Tretarre B, Luce D, Stücker I (2013). Heavy smoking and lung cancer: are women at higher risk? Result of the ICARE study. Br J Cancer.

[5] Elci OC, Akpinar-Elci M, Alavanja M, Dosemeci M (2003). Occupation and the risk of lung cancer by histologic types and morphologic distribution: a case-control study in Turkey. Monaldi Arch Chest Dis.

[6] MacArthur AC, Le ND, Fang R, Band PR (2009). Identification of occupational cancer risk in British Columbia: a population-based case-control study of 2,998lungcancers by histopathological subtype. Am J Ind Med.

[7] Baker EL, Honchar PA, Fine LJ (1989). Surveillance in occupational illness and injury: concepts and content. Am J Public Health.

[8] Chang SS, Kim SY (2000). Contact dermatitis surveillance system in Taejon city: Prevalence of contact dermatitis among the workers exposed to solvents and nonmetallic chemicals through an immediate health examination system in Taejon city. Korean J Occup Environ Med.

[9] Jeong WC, Kwon HJ, Ha M, Roh SC, Kwon BS, Hyun JG, Lee SJ, Lee JM, Kwon JY, Kim JS, Baek NJ, Lee H, Lee KW, Lee SK (2004). Surveillance of work-related carpal tunnel syndrome in Korea. Korean J Occup Environ Med.

[10] Kim SA, Kim JS, Jeon HR, Jung SJ, Kim SW, Lee CY, Ham JO, Yoo JY, Choi TS, Goo HB, Cho MH, Woo KH (2003). Surveillance of work-related diseases in Kumi. Korean J Occup Environ Med.

[11] Kim JI, Kim BG, Kim JW, Chae CH, Yi CH, Kang D, Kim JH, Kim JH, Kim YW, Lee YH, Lee JH, Choi Y, Kim JH, Yun HR, Yoo CI, Jeong BG, Jang TW, Kim YG, Yun DY, Kang JU, Kim JE, Ahn JH, Lee DJ, Jang JH, Lee KY, Song HR, Lee YH, Cho BM (2003). Occupational disease surveillance system in Busan, Ulsan, Kyung-Nam area. Korean J Occup Environ Med.

[12] Bondy SJ, Victor JC, Diemert LM (2009). Origin and use of the 100 cigarette criterion in tobacco surveys. Tob Control.

[13] Rushton L, Hutchings SJ, Fortunato L, Young C, Evans GS, Brown T, Bevan R, Slack R, Holmes P, Bagga S, Cherrie JW, Van Tongeren M (2012). Occupational cancer burden in Great Britain. Br J Cancer.

[14] Driscoll T, Nelson DI, Steenland K, Leigh J, Concha-Barrientos M, Fingerhut M, Prüss-Ustün A (2005). The global burden of disease due to occupational carcinogens. Am J Ind Med.

[15] LaDou J (2004). The asbestos cancer epidemic. Environ Health Perspect.

[16] Hillerdal G, Hendeerson DW (1997). Asbestos, asbestosis, pleural plaques and lung cancer. Scand J Work Environ Health.

[17] Kim HR (2009). Overview of asbestos issues in Korea. J Korean Med Sci.

[18] Park J, Hisanaga N, Kim Y (2009). Transfer of occupational health problems from a developed to a developing country: lessons from the Japan-South Korea experience. Am J Ind Med.

[19] Straif K, Benbrahim-Tallaa L, Baan R, Grosse Y, Secretan B, El Ghissassi F, Bouvard V, Guha N, Freeman C, Galichet L, Cogliano V, WHO International Agency for Research on Cancer Monograph Working Group (2009). A review of human carcinogens–part C: metals, arsenic, dusts, and fibres. Lancet Oncol.

[20] International Agency for Research on Cancer. **IARC: Diesel engine exhaust carcinogenic.** [http://www.iarc.fr/en/media-centre/pr/2012/pdfs/pr213_E.pdf]

[21] International Agency for Research on Cancer: **IARC Monographs on the Evaluation of Carcinogenic Risks to Humans.** [http://monographs.iarc.fr/ENG/Monographs/vol53/index.php]

[22] Blair A, Grauman DJ, Lubin JH, Fraumeni JF (1983). Lung cancer and other causes of death among licensed pesticide applicators. J Natl Cancer Inst.

[23] Alavanja MC, Dosemeci M, Samanic C, Lubin J, Lynch CF, Knott C, Barker J, Hoppin JA, Sandler DP, Coble J, Thomas K, Blair A (2004). Pesticides and lung cancer risk in the agricultural health study cohort. Am J Epidemiol.

[24] Environmental Protection Agency: **Guidance on Cumulative Risk Assessment of Pesticide Chemicals That Have a Common Mechanism of Toxicity.** [http://www.epa.gov/oppfead1/trac/science/cumulative_guidance.pdf]

[25] Lubin JH, Blot WJ (1984). Assessment of lung cancer risk factors by histologic type. J Natl Cancer Inst.

[26] Zheng T, Holford TR, Boyle P, Chen Y, Ward BA, Flannery J, Mayne ST (1994). Time trend and the age-period-cohort effect on the incidence of histologictypes of lung cancer in Connecticut, 1960-1989. Cancer.

[27] Devesa SS, Shaw GL, Blot WJ (1991). Changing patterns of lung cancer incidence by histological type. Cancer Epidemiol Biomarkers Prev.

[28] Zahm SH, Brownson RC, Chang JC, Davis JR (1989). Study of lung cancer histologic types, occupation, and smoking in Missouri. Am J Ind Med.

